# Comprehensive review on sexual dimorphism to improve scalp acupuncture in nervous system disease

**DOI:** 10.1111/cns.14447

**Published:** 2023-09-04

**Authors:** Chaojie Wang, Jiening Wang, Xubo Wu, Tao Liu, Feng Wang, Huanxia Zhou, Chen Chen, Lijuan Shi, Lin Ma, Tiantian Liu, Cancheng Li

**Affiliations:** ^1^ Department of First Clinical Medical College Heilongjiang University of Chinese Medicine Heilongjiang China; ^2^ Department of Rehabilitation Shanghai Seventh People's Hospital Affiliated to Shanghai University of Traditional Chinese Medicine Shanghai China; ^3^ School of Rehabilitation Science Shanghai University of Traditional Chinese Medicine Shanghai China; ^4^ Department of Bioengineering Imperial College London London UK; ^5^ First Affiliated Hospital of Heilongjiang University of Chinese Medicine Harbin China; ^6^ Second Affiliated Hospital of Heilongjiang University of Chinese Medicine Harbin China; ^7^ School of Biological Science and Medical Engineering Beihang University Beijing China

**Keywords:** brain science, innovation, scalp acupuncture, sexual dimorphism, traditional Chinese medicine

## Abstract

**Background:**

With the development of modern medicine, the Traditional Chinese Medicine (TCM) combined with western medicine began to be produced and applied. Scalp acupuncture (SA) as a Chinese medicine based on neurological theory, has a great advantage compared with TCM in the treatment of nervous system diseases.

**Method:**

In this paper, we analyze the physiological and pathological manifestations of sexual dimorphism (SD) to illustrate the necessity of SD treatment. In addition, we review the factors that can affect SD and analyze in physiological structure, function, and pathological neurons. Diseases (pathological basis, pathological manifestations, and incidence) and factors leading to gender differences, which to analyze the possibility of gender differences in SA.

**Result:**

Furthermore, we creatively a new insight of SD‐SA and provide the complete SD treatment cases on the basis of the existing SA in different kinds of diseases including stroke, migraine, attention deficit hyperactivity disorder (ADHD), and depression.

**Conclusion:**

In summary, we believe that it is feasible to improve the clinical effectiveness of SA, which is able to promote the development of SA, and then provides an actionable evidence for the promotion of precision medicine in the future.

## Graphical abstract

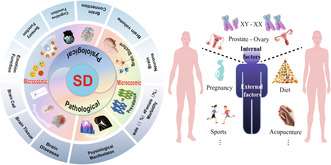

## 1 CURRENT STANDARDS AND RECENT ADVANCES IN SCALP ACUPUNCTURE

Acupuncture and moxibustion are one of the important treatment methods of Traditional Chinese Medicine (TCM). At present, acupuncture and moxibustion therapy has been obtained from the World Health Organization (WHO) traditional medicine strategy data released by WHO.* In 2014–2023, we can find that in the reports of all 129 countries,^1^ 80% of the countries now recognize the use of acupuncture and moxibustion. The main treatment theory is based on TCM, selecting corresponding acupoints and reinforcing & reducing techniques, which can regulate and balance meridians and play a therapeutic role. In addition, ancient Chinese physicians summarized the traditional theory of syndrome differentiation and treatment, which reflected their clinical experience and certain cultural and era characteristics.

Advances in biomedical technology have advanced the field of medical technology, which has made it possible to provide and utilize neuroscience methods from traditional meridian and acupoint selection criteria. It is worth noting that scalp acupuncture (SA) is a method of acupuncture based on brain anatomy and function, which is useful for treating neurological disorders. Despite this, acupuncture and moxibustion have very slow development rates compared to western medicine. Therefore, we review the potential role of emerging brain science theories of sexual dimorphism (SD) in the field of SA in this paper. Furthermore, due to the fact that many theories cannot be effectively applied in clinical practice, we will focus most of our discussion on manipulation in the clinic.

### 1.1 Emergence and present

In 1970, Jiao Shunfa creatively introduced neurobiology into the theory of acupoint selection. Specifically, acupuncture stimulation was performed on this area using the extracranial projection of the cortical localizationism (CL) as a reference, along with the patient's symptoms. Jiao's Scalp Acupuncture (JSA)^2^ has a therapeutic effect on the nervous system, which has also been proven that the curative effect is superior to traditional acupoint selection.

Furthermore, the clinicians discovered that SA can effectively treat the nervous system and non‐nervous system diseases in March 1991.^3^ Additionally, there are more than 10 systematic theories including western anatomy, holography, and the meridian induction phenomenon^4^ currently available about scalp acupoint selection. Among these, non‐ethic Chinese professor Yamamoto developed a new acupuncture method based on Shenting (GC24) and according to clinical experience on scalp points A, B, C, D, E.^5^

The World Health Organization Asia‐Pacific region developed a standard acupuncture nomenclature (Part 2 Re‐ vision [SAN])^†^ in 1984. Specifically, the functional areas of the cerebral cortex are simplified into one line (the meridians and acupuncture points of the head). Subsequently, the standard Manipulation of scalp acupuncture (SMSA) for acupuncture was formulated by the World Federation of Acupuncture Societies in 2006 (GB/T 21709).^‡^ It is defined SA as the manipulation of an acupuncture acupoint (position), and a separate therapy SA was listed as a separate therapy in the formulation of SMSA. Moreover, the state regulatory commission (SRC) and standardization administration of the People's Republic of China (SAC) re‐revised as (GB/T 21709.2‐2021)^§^ on Dec. 2021. As a result, SA has been widely accepted and its promotion has also been regulated by the industry. Finally, the TCM theory profiling in SA is given in Table 1.

**TABLE 1:** The TCM theory profiling in SA.

Year	Name	Author/Unit	Tips
Qin dynasty (221‐207BC)	TSA	Ancient TCM clinicians	Acupuncture theory has 2,500 years
1970	JSA	Jiao Shunfa	The first non‐meridian theory of acupuncture and moxibustion therapy
1991.03	SAN	The SA was recognized from the WHO	First be approved by WHO
2006.07	SMSA	The world federation of acupuncture‐moxibustion societies	SA is a nationally recommended acupuncture operation method (GB/T 21709)
2021.12	SMSA	SRC and SAC	SA has been updated and recommended by the state (GB/T21709.2‐2021)
Our	SD‐SA	This study	Revised

Abbreviations: JSA, Jiao's Scalp Acupuncture; SAC, standardization administration of the People's Republic of China; SAN, Standard acupuncture nomenclature: part 2 revised edition; SD‐SA, Sexual dimorphism of scalp acupuncture; SMSA, Standard manipulations of scalp acupuncture; SMSA, Standard manipulations of acupuncture and moxibustion‐part2: Scalp acupuncture; SRC, The state regulatory commission; TSA, Traditional scalp acupuncture.

### 1.2 Dilemma

Almost 50 years, over 85% of individuals opted for JSA and SAN, with close to 45% of them utilizing JSA.^6^ It is noteworthy to mention that^7^ succinctly delineated the primary acupoint selections for post‐stroke hemiparesis treatment, encompassing SAN, traditional SA (TSA), Zhu's Scalp Acupuncture (ZSA), JSA, and Lin's Scalp Acupuncture (LSA). It is worth mentioning that among the 35 included RCTs that used SA to treat stroke sequelae, 32 used SAN, and 22 of them only used it. The timeline development of SA in TCM theory is given in the Figure 2. Furthermore, the use of the anterior oblique parietal temporal line and the posterior oblique parietal temporal line of JSA and SAN was adopted by investigators, which show that major aspects of SA selection remain subservient to JSA, while others have limited acceptance. Consequently, there exists a need for enhanced advancement and elevated proficiency in its promotion and educational attainment.

**FIGURE 1:**
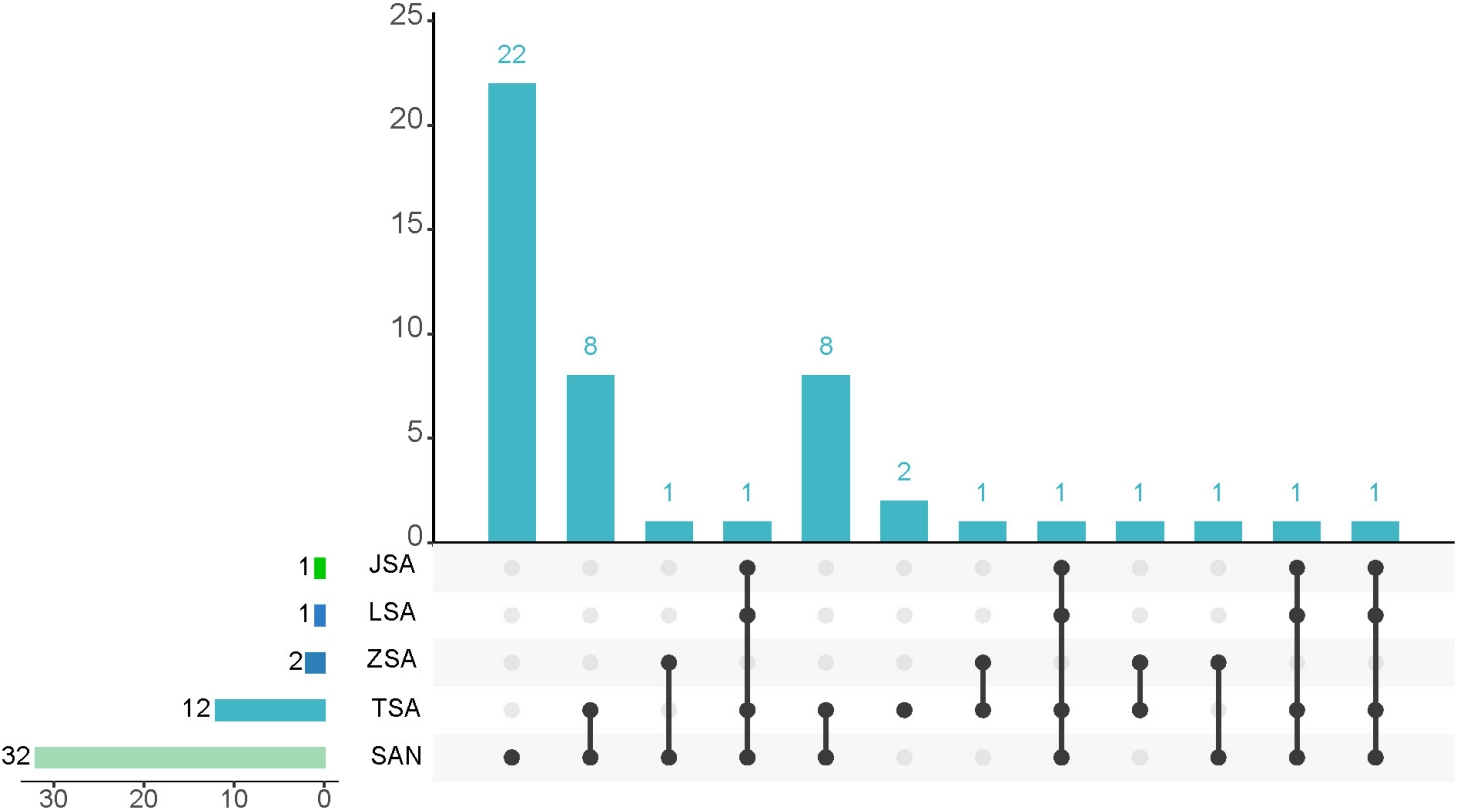
The distribution of acupoints selected for SA in the treatment of sequelae of stroke.

It is worth mentioning that WHO also called them A.1.6 MS6. In addition, JSA was established in the 1970s, and the content of only 16 fields is simple. The main basic theory of CL has undergone some changes with the development of science in recent years. However, JSA (SAN) has not been further revised with the improvement of scientific theory, and the core basic theory and development direction of SA are still lacking. More importantly, neuroscience‐based approaches have yet to be widely used. The current clinical acupuncture operation has attracted attention to further improvement and development.^8^ Therefore, this paper introduces the new theory of SD in brain science into the basic theory of SA based on the current issue. We summarize the studies about SD, and analyze the reasonability and necessity of SD combined with SA therapy. Then, we propose the specific implementation plan of SD‐SA based on current SA treatment options for nervous system diseases. Besides, the distribution of acupoints selected for SA in the treatment of sequelae of stroke is given in the Figure 1.

**FIGURE 2:**
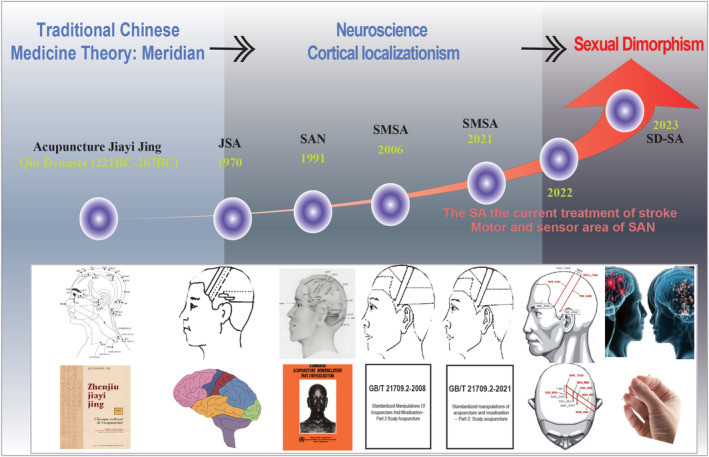
The timeline development of SA in TCM theory. The highlight landmark research in SA research. It is worth mentioning that a more specific explanation of the motor and sensor area of SAN is given in Figure 1, and we reviewed 32 among 35 related studies using SAN.

**FIGURE 3:**
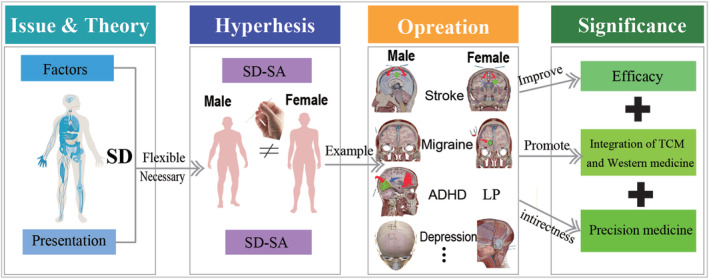
The diagram of our proposed SD‐SA. In particular, we have given more detailed information on the presentation and influencing factors in Figure 4A,B, respectively.

**FIGURE 4:**
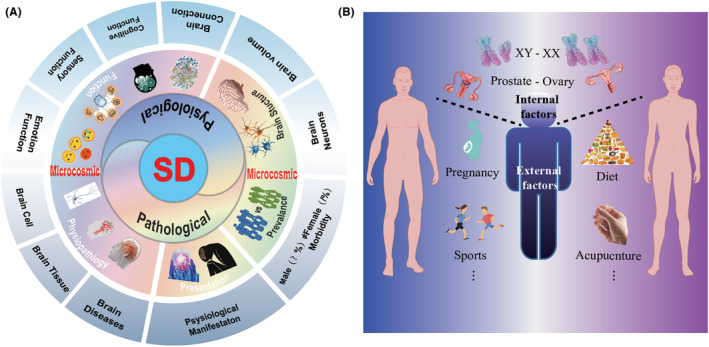
The evidence of supporting SD‐SA. (A) the SD present in neuro. (B) The factors that promote gender differences. It is worth mentioning that gender differences appear in all organs of the body.

**FIGURE 5:**
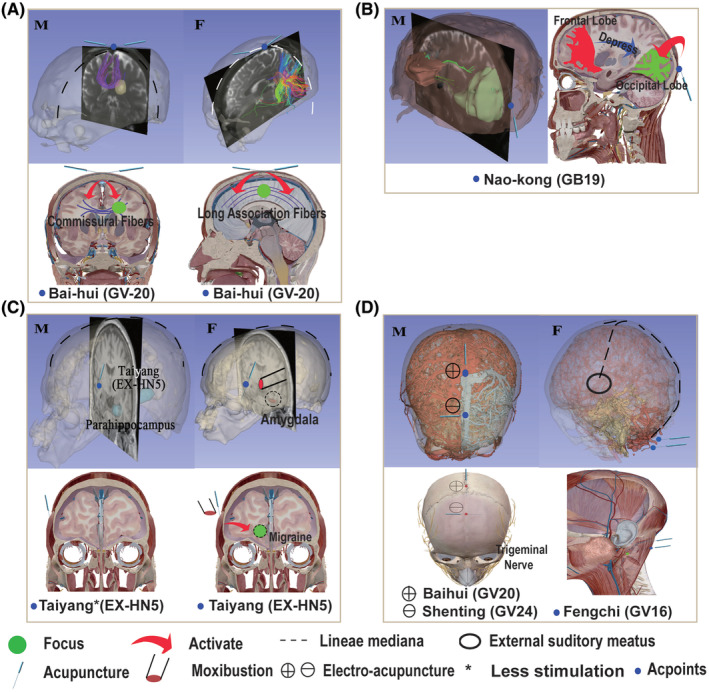
The different neurological diseases for the treatment of SD. (A). A sign of SD‐SA theory on Baihui (GV 20) for stroke patients with basal ganglia infarction (M) A sign of SD‐SA theory on Baihui (GV 20) for male patients in a median sagittal position. (F) A sign of SD‐SA theory on Baihui (GV 20) for female patients in a median sagittal position. (B). A sign of SD‐SA theory on Baihui (GV 20) for male patients in a median sagittal position. (C). A sign of SD‐SA theory on Naokong (GB19) for male ADHD patients. This is the middle of the right eye in the sagittal position. (D). A sign of SD‐SA theory on Fengchi (GV16), Baihui (GV20) and Shenting (GV24) for Depression patients. This Figure shows the arterial blood flow of the whole brain, where red, blue, and yellow are normal blood flow, reduced blood flow, and hyperperfusion, respectively. (M) A sign of SD‐SA theory on Baihui (GV20) and Shenting (GV24) for male patients. (F) A sign of SD‐SA theory on Fengchi (GV16) for female patients. Among them, M and F represent two different situations of male and female.

It has been observed that there exist gender disparities within the divine‐level system based on clinical expertise. Given that the management of divine‐level system ailments varies according to gender, we hereby put forth a hypothesis as a potential solution to address this concern. Thus, we present the concept of SD‐SA, the application of gender‐specific approaches in SA therapy for neurological disorders. Furthermore, we provide evidence substantiating the viability and precise execution of this hypothesis across distinct neurological conditions, culminating in an analysis of the anticipated triumph and tangible ramifications of the proposed theory. Besides, the diagram of our proposed SD‐SA is given in the Figure 3.

## 2 CHARACTERISTICS OF BRAIN SEXUAL DIMORPHISM

The theory of belief formation in classical liberalism emerged in 1850^9^ and reached maturity in 1909 with the elaboration of brain cartography by Korbinian Brodmann.^10^ Nowadays, it is worth mentioning that Brodmann's brain maps have been used in the field of neuroscience. However, the research found the deficiencies and defects of CL.^11^ Further, the SD of the brain and white matter associationist theories improve the development of CL.^12^ Meanwhile, the SD of the brain is the hottest topic worldwide^13^ in neuroscience. It has recently been highlighted in Nature^14^ and Science journal^15^ that gender bias is an issue that has gained public awareness. Biological processes affecting female animal models are significantly influenced by their genetic and hormonal cycles, which still need to be studied to improve the quality of life for both men and women.

### 2.1 Physiological characteristics of brain sexual dimorphism

#### 2.1.1 Brain structure

Several aspects of the brain's anatomy are affected by SD, including the brain volume, cerebral cortex, gray matter, and white matter etc., as well as brain cell development. It has been shown that men have a larger total brain volume (about 9–11%)^16^ than women, regardless of whether they are in childhood, adolescence,^17^ or adulthood.^18^, ^19^ Furthermore, there are also local differences in brain volume between men and women, with the left posterior cingulate gyrus of men having a larger area that is related to language, while the right hemisphere of women has a smaller area related to language.^18^

Besides, it was recently discovered that bisexual brain structure differs not only in volume and cortex, but also in gray matter, white matter, and cerebrospinal fluid.^20^ Male brain putamen gray matter is dramatically larger than female,^21^ while female putamen white matter is larger than male. The white matter in female brains^22^ also exhibits greater connectivity as a sign of neuroanatomical connectivity. There is a difference in brain volume between the cerebral cortex and the prefrontal cortex in adults. Furthermore,^23^ found that a noteworthy revelation wherein the cerebral cortex of male rats exhibited a remarkable magnitude of thickness when juxtaposed with their female counterparts. This noteworthy occurrence was observed across various regions of the brain, including the frontal, parietal, and occipital cortices, as well as within the intricate framework of the corpus callosum, which serves as a vital interconnection between the male and female cerebral domains.

Since the 1970s, neuroanatomical differences between women and men have been gradually detected. During development and maturation, SD in the brain is associated with differences in regional volume, which may be caused by differentiated cell death, neuronal and glial generation, dendritic branching, and synaptic patterning.^24^ It has been concluded that brains are unlikely to be uniformly male or female as a result of these varieties and regional specificity.

#### 2.1.2 Brain function

Due to the differences in the structure of the sex's brains, cognitive abilities, feelings, and emotion regulation of brain function are greatly influenced. The difference between men and women in cognitive function can be seen in spatial tasks, and in language fluency and memory.^25^, ^26^ In addition, adult males have stronger functional connectivity to the sensory‐motor cortex, whereas females have stronger connections to the default mode network.^19^ For the purpose of reducing the influence of social factors on SD,^27^ explained that marmoset females perform worse than males in reverse learning tasks. The reason for this difference is that the neural network connectivity in female brains is significantly lower than that of males.

Subsequently, Bessinis^28^ found significant differences in visual neurochemistry for retinal, visual cortex tryptophanergic, and dopaminergic effects in SD. Thus, there may be different mechanisms for visual processes between the sexes. Additionally, differences were identified in the right hemisphere of the Heschl's gyrus (HG) and the posterior superior temporal gyrus (pSTG) of males and females, brain regions important for auditory processing, and more specific differences in auditory function.^29^

In,^30^ the authors also found that women had larger ventromedial prefrontal cortex, right orbitofrontal cortex, bilateral basal ganglia, and nearby white matter than men. Moreover, self‐reported emotion regulation strategies, expression of positive emotions, impulsive emotions, and non‐somatic cognitive anxiety is correlated with ventrome‐ dial prefrontal cortex development. As a result, physiologically normal differences in brain volume may play a role in emotion regulation and individual differences in emotion.

### 2.2 Pathological characteristics of brain sexual dimorphism

Diet has a significant impact on several issues, including organ function, immune function, disease incidence, dis‐ ease state, and survival. Additionally, there are differences in physiological and pathological changes between the sexes. There are many pathological processes, disease incidence, prevalence, and mortality parameters associated with SD in mammals.^31^, ^32^ Experiments with diet‐induced obesity found sex‐and age‐dependent changes in the adiponectin/leptin ratio.^33^ It is noteworthy that obese mice react differently to obesity and metabolic disorders based on gender and age. It may be possible to develop more effective obesity treatments based on these results, which will contribute to a better understanding of the metabolic effects of obesity.^34^

Due to the different immune responses, men and women have foreign and self‐antigens, as well as their innate and adaptive immune systems. Thus, there is a gender bias of disease prevalence in neurological disorders, infectious diseases, inflammatory diseases, cancer, etc.^35^, ^36^ A majority of autoimmune diseases occur in women, accounting for 80% of the incidence of these diseases, which include Sjogren's syndrome, systemic lupus erythematosus, scleroderma, and myasthenia gravis.^37^ Furthermore, there are autoimmune diseases including bladder cancer, lung cancer, and other cancers of the reproductive system.^38^ In addition, there are sex differences in susceptibility to various infectious diseases.^39^, ^40^ Sex steroid concentrations decline more rapidly with age in women than in men, which also predisposes women to an abnormally chronic low‐grade proinflammatory state.^41^, ^42^ However, pathogen‐associated lesions, including delayed clearance, were associated with male‐biased infection.^39^

Recent studies have shown that sex‐differentiated microglia cells play a key role in ischemic stroke,^43^ the studies suggest that molecular mechanisms of microglial activation and phenotypic polarization may be influenced by sex in CNS Corruption of system response. In addition, prebiotics also has an effect on gender differences in intestinal and blood–brain barrier dysfunction in stress anxiety and depression. Furthermore,^44^ suggests that intestinal flora, intestinal and blood–brain barrier, and inflammatory responses may mediate the protective effect of prebiotics on anxiety‐like behavior in female mice. Furthermore, Andrew et al.^45^ proposed that sexually dimorphic estrogen perception in skeletal stem cells plays a controlling role in bone regeneration, and that skeletal stem cells (SSCS) of female mice are directly affected by estrogen signaling controls, which may improve bone fracture repair. J.M. Newton using X‐ray fluorescence microscopy and Fourier transform infrared spectroscopy imaging found that patients with the same size near‐infarct core but different metabolic markers in SD surrounding.^46^ This may present alternatives for improved functional recovery in females in the early post‐stroke phase, as the elevated levels were 1.4 times higher. Gender disparities in peripheral immune responses to neurodegeneration subsequent to ischemic stroke have additionally been noted, contributing to variations in post‐stroke outcomes. Notably, male mice subjected to splenectomy displayed reduced infarct areas, whereas their female counterparts did not manifest a similar response.^47^

Furthermore, there are numerous biological factors that contribute to the fact that women live longer than men in the world, including changes in sex steroid concentration and diploidy of the X chromosome.^48^ The maximum life expectancy is 75.4 and 80.7 years for male and female, respectively.** Moreover, the results show that the females self‐estimated expectations (1.7 years) are more close to actual expectations compared with the male (7 years).^49^

### 2.3 Affect factors of brain sex dimorphism

Besides, it is pointed out that 30% of nerve cells differentiate according to sexual preference in.^50^ Furthermore, it has also been shown that most human organs, including the muscles, adipose tissue, liver, immune system, intestine, kidney, and bladder are also affected by SD.^51^, ^52^

SD is strongly associated with internal factors including sex genes and hormones.^53^, ^54^ Whereas, clinical re‐ search has found that external factors also have an essential impact on the development of SD in the brain, including maternal nutritional status during pregnancy and adulthood,^55^ exercise intensity and cognitive level.^56^ Further‐ more, sex is a biological characteristic that is determined by an individual's chromosomes, reproductive organs, and sex steroid levels. It influences immune responses to various antigens, including those from fungi, viruses, bacteria, parasites, and allergens. Gender, on the other hand, encompasses societal and cultural factors that shape an individual's behaviors and activities. It is important to distinguish between sex and gender when considering their effects on treating diseases and keeping health.^35^

Indeed, it is worth mentioning that different genders respond differently to stimuli. The authors of the study cited herein^57^ found that XY and XX neurons have different sensitivities to damage in the culture environment. Another study cited herein^58^ found that male and female rats' intestinal tracts had dissimilar responses to malignant stimuli. It is worth mentioning that capsaicin can cause harmful stimulation to the colon of rats. Male rats with an activated defecation center in the brain and spinal cord have an increased motility response to the colon, but it has no effect on the colon of female rats. Furthermore, the research cited herein^45^ showed that male brains responded significantly better to repetitive transcranial magnetic stimulation (rTMS) than female brains in patients with severe depression. Meanwhile, the sensitivity of the trigeminal nerve to pain is also different,^59^ women are more susceptible to pain than men.^60^

At the same time, the study found that there are differences in the efficacy of the same treatment regimen be‐ tween the sexes. The study referred herein^61^ found that men and women respond differently to aspirin, which has a beneficial effect on myocardial infarction, but not on stroke prevention. Besides, aspirin consumption among women was associated with a reduction in stroke risk, but not myocardial infarction. There are more studies have demonstrated the importance of SD in treating diseases.^62^, ^63^, ^64^

It is worth mentioning that acupuncture and moxibustion is an external treatment methods of TCM and also a neuroregulatory therapy of western medicine. It is safe, easy to operate, has a wide range of diseases, and is highly accepted by patients.^65^ In recent 20 years, there are more than 100 clinical trials and mechanism research that have proven acupuncture efficacy and mechanisms of curing neurological diseases.^66^, ^67^ Specifically, studies have proven that acupuncture is a mechanical stimulus that can activate local cell functions and neuroreceptors. It also regulates the release of related biomolecules (peptide hormones, lipid hormones, neuromodulators and neurotransmitters, and other small and large biomolecules) in the microenvironment, where they can affect each other and further activate the neuroendocrine‐immune network to achieve holistic regulation.^68^

However, most research studies focus on examining and studying the causes and manifestations of acupuncture in treating diseases, which cannot directly provide constructive solutions for the treatment. Besides, it has been possible to improve the curative effect by using combined theory apart from the physiological structure and function of the brain, there are also significant differences in the incidence and pathological changes of brain diseases in males and females. Therefore, we further conclude that there is significant SD in the incidence and pathological changes of brain diseases, including cerebrovascular disease stroke, central nervous system disease migraine, brain development disease, attention‐deficit hyperactivity disorder, neuropsychological disease, and depression, etc. Besides, we introduce the concept of SD in the treatment. The evidence supporting SD‐SA is given in Figure 4.

## 3 TREATMENT

At present, many basic experimental results cannot directly provide constructive solutions for the treatment of dis‐ eases. Therefore, we introduce the concept of SD in the treatment of different nervous system diseases and current clinical practice, and directly provide solutions for the clinical treatment of SA and related diseases, including cerebrovascular diseases, central nervous system diseases, nervous system diseases, developmental brain disorders and neuropsychiatric disorders. Additionally, we present clinical treatment options for SD‐SA in 4 different diseases in the Figure 5.

### 3.1 Stroke

A global study on the incidence and mortality of neurological diseases between 1990 and 2015 found that stroke incidence and mortality are higher for men than women.^69^ Additionally, the mortality rate of women within 3 months after stroke is lower than that of men. However, the functional damage sustained by women is greater than that of men.^70^ Apart from that the functional score of female stroke patients is substantially lower than that of male stroke patients, even after adjusting for age, clinical manifestations, and other confounding factors.^71^, ^72^

SA is an effective therapy for stroke as it stimulates damaged brain tissue, stimulates undamaged brain tissue, and compensates for the lost function of damaged tissue.^7^, ^73^ Generally, Baihui (GV20) is chosen by stroke patients as the acupoint at the intersection of the two ear tips on the competent blood vessel, which corresponds to connectivity of the corpus callosum in males is stronger in the hemispheres,^74^ while in females it is between the hemispheres. Therefore, SD‐SA can be used in the treatment of stroke, especially basal ganglia infarction. For example, acupuncture for Baihui (GV 20) in male patients can use the needle tip along the GV (sagittal plane) to better activate the brain's commissural fibers. However, in female patients, a vertical GV (see coronal plane) approach resulted in better activation of the long commissural fibers of the brain.

### 3.2 Migraine

Brain Migraines are a common neurological disorder. Moreover, in the research studies cited herein,^75^, ^76^ the authors introduced that the changes in the structure and brain function of multiple brain regions are significantly different between men and women. Fur‐ thermore, a study using high‐field magnetic resonance imaging (fMRI) found that women with migraine had specific changes in brain structure in the insular and precuneus regions, while men had specific changes in the parahippocampus change.^77^ Additionally, male migraineurs are more likely than female migraineurs or healthy subjects to be more sensitive to painful stimuli, and women respond negatively to stimuli.^78^

SA has been proven^79^ effective in treating migraine. Specifically, the treatment points are mainly Baihui (GV20), Fengchi (GB20), Fengfu (GV16), bilateral Taiyang (EXHN5), and bilateral Hegu (LI4). Moreover, acupuncture can play a significant role in amygdala‐insula‐superior temporal gyrus connectivity^80^ in patients with migraine. Therefore, due to the differences in the brain function performance of male and female migraine patients and the mechanisms of SA in treating migraine, SD should be distinguished during SA treatment. Specifically, less stimulation should be used for male patients, while for female migraine patients, pain relief should be used. In addition, the amygdala and parahippocampus of female patients with migraine have obvious responses to thermal stimulation.^77^ Therefore, SA can be combined with moxibustion, red light, and other thermal therapy techniques to treat severe female migraine patients, which can achieve the alteration of brain functional connectivity and improve curative effect.

### 3.3 Attention‐deficit hyperactivity disorder

Attention‐deficit hyperactivity disorder (ADHD) is a chronic nervous system development disorder, which has a far greater number of males than females.^81^ The symptoms include inattention, hyperactivity, or impulsivity, and behavioral and learning problems are more severe in males.^82^

Diffusion tensor imaging (DTI) as an effective electrical imaging technique has been used efficiently to detect the microstructure of white matter tracts in ADHD. In the study referred to herein,^83^ the authors found that male children had lower white matter structural heterogeneity and functional connectivity than females. Specifically, male children have a thinner right frontoparietal cortex and a thicker occipital cortex. The degree of cortical changes is positively correlated with the severity of the disease. The abnormal brain development in children with ADHD, mainly in the occipital region. Currently, acupuncture is an effective treatment for ADHD,^84^ with Baihui (GV 20) and Si shencong (EXHN1) being the most commonly the central gyrus of the corpus callosum.

The pathological changes and mechanisms of ADHD children's brain^85^ show that SA can effectively promote the secretion of extraoccipital amino acids, and the treatment of boys should stimulate the occipital lobe of the right brain hemisphere. The lateral superior border of the right extraoccipital eminence adds brain space (GB19), which can increase the excitability of the right occipital lobe and promote the growth and development of the right frontooccipital tract. In conclusion, there is a delay in ADHD^86^ that allows the right hemisphere to develop normally.

### 3.4 Depression

Compared to men,^87^ adolescent and adult women have a higher rate of depression, with male depression patients having a higher suicidal tendency. Despite the tendency for female depression patients to start early,^88^ females have lower depression symptoms. Besides, in,^89^, ^90^ the authors point to changes in brain structure and function in SD patients with depression.

There is evidence that abnormal cerebral blood flow may be causing depression symptoms in patients. In,^91^ the left prefrontal cortex of men showed a significant drop in cerebral blood flow under stress conditions. Nevertheless, women's ventral striatum, insula, and cingulate cortex blood flow increased under the same conditions. In addition, women with depression show that the painful symptoms may result from excessive perfusion of the emotional brain area of the amygdala.^92^

Currently, depression is significantly treated with acupuncture at scalp points Baihui (GV20) and Yintang (GV29).^93^, ^94^ It is mentioned that electroacupuncture can significantly influence blood flow velocity within the cerebral cortex.^95^ Furthermore, the use of electroacupuncture^96^ also had a significant impact on reducing blood pressure, thereby achieving the purpose of improving cerebral blood flow. Furthermore, stimulating the trigeminal nerve can effectively dilate cerebral blood vessels,^97^ increase cerebral blood flow, and improve the ischemic state, so acupuncture Baihui (GV20) and Shenting (GV24) which needle tip along the GV and tip vertical GV, respectively. And a group of electroacupuncture sessions with them, so as to better stimulate the trigeminal nerve and restore the slowed cerebral blood flow in the anterior hemisphere of male depression. Compared with men, female patients with depression are in a state of cerebral hyperperfusion in the amygdala and insula. SA and electroacupuncture are not suitable, because they will further promote cerebral blood flow. We can choose acupoints like Fengchi (GB20) other than SA, and the acupuncture at the Fengchi point on both sides can stimulate the vertebrobasilar artery, which improve the state of the circle of Willis and promote the normal distribution of cerebral blood flow,^98^ while stimulating the parasympathetic nerve can reduce the pressure of blood flow and improve the state of the brain.^99^ Furthermore, the data we used for the original images are given in the Appendix S1.

## 4 FUTURE RESEARCH AND DIRECTIONS

It is critical for innovative clinical treatment of TCM to be clinically effective. The improvement of the clinical curative effect is attributed to the innovation and perfection of TCM clinical treatment theory. However, the current innovative methods of TCM treatment is mainly guided by the clinical experience of famous veteran TCM practitioners, who have a unique understanding of the pathogenesis of certain diseases, and TCM syndrome differentiation and treatment. In addition, the goal of these clinical studies is to find supporting and experimental evidence that their treatments are effective. Indeed, many clinical mechanisms have greatly promoted medical understanding of diseases and therapies, mainly providing theoretical references for the clinical treatment of diseases. Unfortunately, many basic research results have not been directly and effectively translated into clinical practice, which has also led to the loss of the profound significance of the research. Therefore, we mainly focus on formulating a reasonable clinical application plan on the basis of theoretical research, providing an interactive channel for theory and practice.

Combining JSA with nervous system theory mainly develops new methods of acupoint selection, which effectively improves the curative effect of SA on nervous system diseases. Due to the changes of the times and the development of science, the theories used by JSA have also undergone further development and changes. We comprehensively review the necessity and flexibility of SD‐SA from the current situation of SD and its influencing factors, and also give specific operations for SD clinical treatment operations, which can effectively prove the clinical feasibility of SD‐SA to improve SD‐SA efficacy.

Acupuncture, as a safe, convenient, and economical treatment method, has been recognized and promoted by the international community.^100^ Nevertheless, the main theory of acupuncture (i.e., meridians) is fundamentally different from that of western medicine, which also brings many difficulties to western clinicians in learning and using acupuncture. Especially, acupuncture treatment programs can be individually designed according to each patient's condition, which can better meet the needs of patients. Not all doctors are able to master them proficiently.

Acupuncture combined with neuroscience has enriched its own theory, and at the same time improved the curative effect, which has been recognized by more people. Since SD‐SA refers to more nervous system theories, it can promote the integration of Chinese and Western medicine and help more doctors learn and use acupuncture. The SD‐SA provides a method derived from clinical practice and the program is redefined, which will help SD‐SA be widely accepted, disseminated and clinically implemented by clinical experts, and will greatly promote the awareness and promotion of SA technology. However, although acupuncture is widely used in the treatment of various diseases, its therapeutic effect lacks scientific evidence support, and more research is needed to verify its effectiveness.

In sum, the acupuncture technology has been recognized all over the world for its excellent curative effect, but the research on the mechanism and standardization of acupuncture and moxibustion treatment is still in its in‐fancy. Finally, it is worth mentioning that although acupuncture originated in China, it needs multi‐dimensional knowledge to promote its further development, so it requires the joint efforts of acupuncture scholars all over the world to rapidly promote the development of acupuncture techniques.

In this paper, we argue that SD‐SA can play an important role in precision medicine. In precision medicine, the concept of second childhood can be used to develop personalized treatment plans for different patients. Specifically, SD serves as an important factor (such as physiology and psychology) in medical research and treatment, which may affect the effect of treatment. Besides, recent research began to move away from the ‘one‐model‐fits‐all’ approach to treating disorders into 4P Medicine^101^, ^102^ and dynamic medicine.^103^ However, these models need to take a long time to develop due to limited drug resources, and the emergence of new technologies will also enable optimization and personalization of SA for various neurological diseases. Therefore, the promotion of SD‐SA therapy will promote the innovation and progress of SA acupoint selection, and provide operation and assistance for the transformation from evidence‐based medicine to precision medicine. In short, the new theory promises to improve SA results.

## 5 CONCULSION

Overall, in this paper, our contributions are mainly as follows:

- We comprehensively expounded the differences between the sexes in the physiology and pathology of the god‐ level system, and reasonably put forward the hypothesis of SD‐SA to improve the efficacy of SA.
- The concept of SD in the nervous system in the treatment theory of SA is further introduced in this paper, which enriches the theory of acupuncture and moxibustion and promotes the integration of Chinese and Western medicine. At the same time, SD is also potential contributors to the precision protocol from standardized solutions, which will benefit the development and promotion of precision medicine.
- We discuss the need for clinically effective and innovative clinical treatments of SD‐SA in TCM, which effectively promotes the combination of clinical and basic research, but we must also admit that a large amount of evidence is still needed to prove the validity of the sub‐innovative theory.

## 6 LIMITAION AND FUTURE WORK

In this paper, although we give a specific treatment case for SD‐SA, the real purpose is not to promote the plan. On the contrary, in order to emphasize the importance of SD‐SA and the specific implementation cases under the guidance of this theory. There is no doubt that the mechanism of SA therapy is complex. Specifically, the mechanism of action of SA is complex. To prove the effectiveness of SD‐SA, more clinical data are needed for research. More specifically, we did not focus on a specific method of SD‐SA, but hoped that the existing basic research could promote the development of SA, control the two groups of patients with only gender differences, and control each other. Using the identical SA therapy, juxtapose the therapeutic outcomes of the two cohorts post‐treatment. Once a positive outcome is ascertained, subsequently substantiate the dependability of SD based on the clinical treatment protocol. Furthermore, by means of scrutinizing the pathological distinctions between male and female patients, discern an untapped SA treatment regimen, thereby refining the disparagement in treatment efficacy pertaining to gender.

In the last, acupuncture technology has been recognized all over the world for its excellent curative effect. How‐ ever, the mechanism and standardization research of acupuncture and moxibustion treatment has not attracted more people's attention. The development of acupuncture and moxibustion treatment requires the joint efforts of more acupuncture and moxibustion scholars.

## AUTHOR CONTRIBUTIONS

Chaojie Wang, Tiantian Liu, & Cancheng Li contributed to the study concept, design, and image data processing. Chaojie Wang, Tao Liu, & Cancheng Li performed the drafting of the article. Jiening Wang, Xubo, Wu, Huanxia Zhou, Chen, Lijuan Shi, & Lin Ma contributed to the study concept, and revising of the article for intellectual content. Tiantian Liu, & Feng Wang provided financial support.

## FUNDING INFORMATION

This work was supported in part by the National Natural Science Foundation of China (Grant No.81973930) and Pudong Area “National Comprehensive Reform Pilot Zone of Chinese Medicine Development”. Construction Project (PDZY‐2022‐0702).

## CONFLICT OF INTEREST STATEMENT

The authors have no conflict of interest to declare.

## Supporting information


Appendix S1

## Data Availability

The data and software code that support the findings of this study are available from the Appendix S1.

## Endnotes

*
https://www.who.int/publications‐detail‐redirect/9789241506096

^†^

https://apps.who.int/iris/handle/10665/207637

^‡^

https://webstore.ansi.org/standards/spc/gb217092008‐1683654

^§^

https://www.gbstandards.org/GB_standard_english.asp?code=GB/T%2021709.2‐2021

**
http://www.cbs.nl/
