# Supplementary material for: Comprehensive review on sexual dimorphism to improve scalp acupuncture in nervous system disease
Source: CNS Neurosci Ther. 2023 Sep 4;30(1):e14447. doi: 10.1111/cns.14447 (PMC10805401; doi:10.1111/cns.14447)
Supplement: Supplementary file 1 — Appendix S1 [file CNS-30-e14447-s001.pdf]

supplementary Information

“Comprehensive Review on Sexual Dimorphism to Improve Scalp Acupuncture in Nervous System Disease”

Chaojie Wang, Jiening Wang, Xubo Wu, Tao Liu, Feng Wang,
Huanxia Zhou, Chen Chen, Lijuan Shi, Lin Ma, Tiantian Liu*, Cancheng Li*

In the appendix, we provide the original sample data in this paper, which are all derived from actual patients in the clinic. Specifically, A , B , a nd C s how D TI a nd m ri o f b rain fi ber tr acts, an d D sh ows CT A of cerebral vessels. According to the patient’s original image data, import the data into 3Dslicer, and make necessary modifications a nd i llustrations a s n eeded. W hat’s m ore, w e m ade o ur r esearch d ata p ublicly a vailable on https://figshare.com/account/projects/170928/articles/23580297.
